# Space allowance impacts behavior, productivity, reproductivity and immunity of sheep—a review

**DOI:** 10.1007/s11250-023-03615-2

**Published:** 2023-05-18

**Authors:** Mohamed I. El Sabry, Lebogang E. Motsei, Ibrahim I. Abdel-Mageed, Obaida Almasri

**Affiliations:** 1 Department of Animal Production, Faculty of Agriculture, Cairo University, 6 El-Gamma St, 12613 Giza, Egypt; 2 Department of Animal Science, Faculty of Natural and Agricultural Science, North-West University, Mmabatho, 2735 South Africa; 3 General Commission for Scientific Agricultural Research, Damascus, Syria

**Keywords:** Sheep population, Space allowance, Production system, Wool, Growth rate, Milk yield

## Abstract

Sheep is an important producing animal in subtropical and arid regions; however, sheep farming practices and welfare standards are still not well established. To move to either intensive or intensive sheep production, stocking density (animal/area, SD) is a significant factor that influencing the welfare and productivity of animals. However, there are discrepancies in space allowance standards for wool, meat, and dairy sheep at different stages. Thus, this review article sheds light on (1) the geographical distribution of wool, meat-type, and dairy sheep populations; (2) the effects of interaction among space allowances, housing systems, and group size on the social, feeding, and aggressive behaviors and human-sheep contact; (3) the effects of space allowance on wool, growth performance, and milk production of sheep; (4) the relationship between space allowance and reproductive performance; (5) the effects of stocking rate on immunity; and (6) suggestions to mitigate the stress and deleterious influences of SD on the productivity of sheep. In conclusion, the larger space allowance with access to an outdoor yard can improve social and feeding behaviors, meat and milk yield, and wool quality. Moreover, ewes are more sensitive to SD, so they should receive an adequate space allowance at each stage. The changes in behavioral responses of each sheep breed refer to their different requirements. Therefore, there is a need to determine the impact of housing aspects, especially space allowance and enrichment tools on the productive performance and welfare indices of sheep for implementing welfare-economic standards for sheep production.

## Introduction

The sheep (*Ovisaries*) is one of the first animals that have been domesticated (11,000 BC) due to their appropriate characteristics for domestication such as small body size, adolescence, high productivity, and a variety of products, including wool, meat, skin, and milk (Joy et al. 2020; Ralph Clark et al. 2021).

The stocking density (SD) is the number of animals that grow in a specific area. Since the welfare-cost balance has become a significant aspect of animal production, the optimal space allowance for domesticated animals and birds becomes not only a matter of mass production but also animal health and welfare issues (El Sabry and Almasri 2022; 2023. Space allowance ultimately determines behaviors and productivity that animals will be able to perform and for how long they perform them. Because behavior patterns of feeding, drinking, excretion, and resting activities are critical for the growth of animals, locomotion, social, and self-grooming behaviors impact on health and welfare status of animals (Petherick 2007; El Sabry et al. 2022).

Therefore, the effects of SD/space allowance on behavior, wellbeing, and productivity of sheep have been studied under different production systems. However, the results and recommendations showed a kind of contradiction. This contradiction can be due to the interaction of other experimental factors such as the genetic background of the breed (Abdel-Rahman et al. 2008), size of groups (Kim et al. 1994; Kleeman et al. 2006); Leme et al. 2013;Rovira 2013; Avero’s et al. 2016), and different housing systems (Casamassima et al. 2001; Caroprese et al. 2009).

Therefore, this review sheds light on the following: (1) the geographical distribution of the wool, meat-type, and dairy sheep populations; (2) effects of interaction between SD and size group on the behavior and welfare indices; (3) effects of SD on the yield and quality of wool, meat, and milk; (4) the relation between SD and reproductive performance; (5) the effects of SD on immunity; and finally, (6) how to lessen the stressful effect of high SD on the sheep.

## Geographical distribution of the wool, meat-type, and dairy sheep populations

**Fig. 1:**
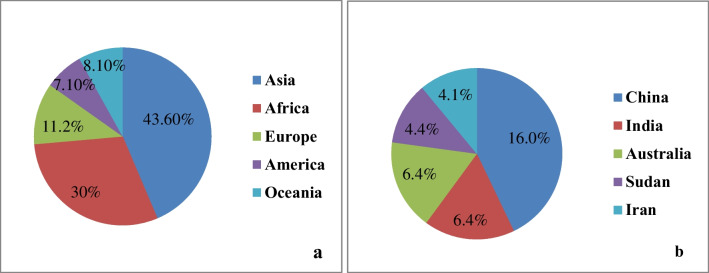
The distribution of sheep populations in different continents (**a**) and countries (**b**)

The total size of the sheep populations all over the world is about 1.26 billion head (FAO 2020), and most of them are distributed in Asia and Africa (Fig. 1). Sheep live in large groups on grazing land. They can rely on low inputs in terms of feed and water, e.g. water footprint of sheep is lower than this of cows (El Sabry et al. 2023). They also are productive under intensive production systems (Joy et al. 2020). Thus, sheep are a favorite animal in rural and Bedouin communities. Moreover, farming sheep is an important activity for food security in many countries (Skapetas and Kalaitzidou 2017; Hegde 2019; Mazinani and Rude 2020). However, the contribution of sheep milk to global milk production is about 3.2%, which is significantly lower compared to the contribution of dairy cattle (83.1%) and buffalo (13.1%) (Pulina et al. 2018). This can be due to the seasonality production of sheep (Albenzio et al. 2016).

China is the leading producer of greasy wool, contributing 19.4% of the total world production, followed by Australia, New Zealand, the UK, and Iran (Skapetas and Kalaitzidou 2017; Pulina et al. 2018). The global production of sheep milk, meat, and wool is 10.6 Mt, 9.9 Mt, and 1.8 Mt, respectively (FAO 2020). Asia is the largest producer of sheep meat (48.6%), followed by Africa (20.2%) and Europe (13.9%).

Sheep meat consumption comes in fourth place after pork, poultry, and cattle (Mazinani and Rude 2020). The global sheep-meat consumption average is 1.7 kg/capita, but it varies from 17 kg/capita in Oceania to 0.7 kg/capita in North America (Montossi et al. 2013). The leader in sheep meat production country is China with 2,080,000 tonnes, which constitutes 50.8% of Asia and 35.9% of the global world’s meat production. On the other hand, China is the first importer and consumer of sheep meat. EU is the second-largest producer and importer of sheep meat (Skapetas and Kalaitzidou 2017).

Also, Asia is the main sheep milk producer (45.6%), with remarkable amounts in China and Turkey, followed by Europe (29.0%) and Africa (24.5%). Additionally, there is a small but growing in milk production in the North and South Americas (0.9%) and Oceania (< 0.1%).

Sheep milk is the richest in fat, solids, and minerals compared with cow and goat milk, which makes it ideal for producing high-quality cheese (Watkins et al. 2014). Also, from a medical viewpoint, sheep milk is more healthy for people who have a kind of allergy against cows’ dairy products (Mazinani and Rude 2020). But, it is worthy to note that some tolerant patients to cow milk are allergic to sheep milk (Bernard et al. 2021).

**Fig. 2:**
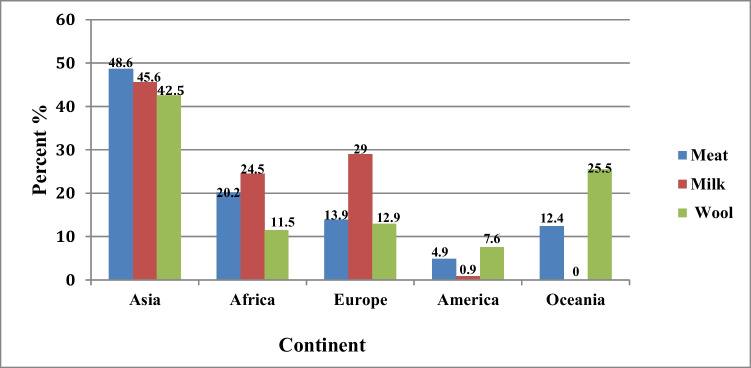
Distribution of sheep meat, milk, and wool production worldwide

The global market of sheep-milk cheese accounted for approximately US$374 million. Italy leads the exportation of sheep-milk cheese, which is followed by France with a market share of 36% and 20%, respectively. On the other side, the USA and Germany are the main importers of sheep cheese with a share of 42 and 41%, respectively (Mazinani and Rude 2020). Wool has been of importance throughout human history and was the first product to warrant international trade. Wool products, including cloth, carpeting, and blankets represent only 3% of world wool production, but it is crucial to the economy in developing countries (Mazinani and Rude 2020). There are a few high-quality wool-producing breeds, including Lincoln, Merino, and Karakul. The average sheep wool production is between 2 and 5 kg/head/year (Robards 1979), but wool production of some breeds, e.g., Lincoln and Merino can reach up to 22 g dry wool/day (Hogan et al. 1979). In recent decades, wool production and its trade markets suffered from an economic crisis due to the increased use of synthetic fibers, and alteration in consumers’ awareness of animal welfare (Hustvedt et al. 2013). Finally, the distribution of sheep milk, meat, and wool production in the world is summarized in Fig. 2.

### Impact of SD on behavior and welfare indices

Animal welfare is an indispensable component of modern animal production systems (Le Neindre et al. 2017). However, the capacity of sheep to cope with harsh environmental conditions led to a misconception that sheep do not need a welfare assessment (Sevi et al. 2009). However, improving the welfare status of animals can be a sustainable strategy for enhancing the animal’s well-being and health. Consequently, the ecosystem and consumers can benefit from the reduction of chemicals and the spread of antibiotic-resistant bacteria.

Space allowance is an environmental factor that directly and indirectly impacts the animal’s performance under intensive systems. Petherick (2007), El Sabry et al. (2022), and El Sabry and Almasri (2022) mentioned that determining the required space allowance for an animal with welfare in mind is a challenging awareness and cost efficiency issue. Moreover, other environmental factors and animals related factors such as physiological status, production systems, construction materials, and facility design can affect the accessibility of animals to fodders and drinkers. From a welfare point of view, optimizing SD for sheep will help them to express their species-specific behaviors, while stressful conditions can negatively alter their physical responses and behaviors (Caroprese et al. 2009).

In rams, Engeldal et al. (2013) evaluated the effect of three space allowances (3.2, 1.6, 0.8 m^2^/ram) on the social behavior of Barbados Blackbelly Cross (BC), Local Garut (LG), and Composite Garut (KG) breeds (age 2–3 years and average 32-kg body weight (BW)). They reported that a large space allowance of 1.6 or 3.2 m^2^/ram increased exploratory, locomotion, and standing activities compared to those housed on the low space allowance (0.8 m^2^/ram), while space allowance did not affect self-care, agonistic, and mating behaviors. Logically, the decrease in movement in the smaller space of 0.8 m^2^/ram is due to physical constraints on the rams’ movement.

Regarding the effect of breed on social behavior, Engeldal et al. (2013) found that the KG breed had the lowest mating and agonistic behavior and the highest self-care and aberrant behavior compared to other BC and LG breeds. On the other hand, there were no significant differences in exploratory, locomotion, and standing among the studied breeds. They suggested that variation in the behavior of sheep is associated with the genetic background of breeds. Moreover, it was noticed that aggressive behavior displayed by different sheep breeds significantly differs. Therefore, it is worthwhile to determine the sheep space limit need for each sheep breed.

In pregnant Norwegian Dala ewes, Bøe et al. (2006) studied the effect of different space allowances (0.5, 0.75, and 1.0 m^2^/ewe) on movement and resting behaviors of 24 medium-sized (aged 2 to 6 years, 80 to 85 kg BW). The standing, total lying time, synchronization of lying, lying close to the wall, and lying in the lying area (% of lying observation) were significantly lower when the space allowance was reduced from 1.0 to 0.5 m^2^. While, displacements, lying close to one or more ewes (lying in the lying area %), and walking (% of total observations) increased in the smaller space allowance.

One- to eight-year-old pregnant Latxa ewes showed more negative social interactions in a small space (1 m^2^/ewe) compared to those in a large space (2 or 3 m^2^/ewe), in Spain, (Avero’s et al. 2014). This increase in negative social interactions might result from the physical proximity of neighboring sheep in the smaller space. Also, ewes in a small floor space spent less time moving and more time at the fodder (*P* < 0.05) than ewes stocked in a large space. Also, in Norway, Dalholt (1985) found a higher number of displacements in the case of reducing the space allowance from 0.86 to 0.69 m^2^/ewe.

In early lactating Comisana ewes (BW 54 kg), Caroprese et al. (2009) suggested that a space allowance of 3m^2^/head was sufficient space for increasing ewes’ activity, including eating, ruminating, walking, playing, and resting. Furthermore, they reported that space restriction resulted in less movement, but a more rigid social relationship and aggressive interactions. It can be driven that contradiction between the walking time and other behavior indices results in the abovementioned examples, which can be due to the different space allowances or production stages.

In addition, Vik et al. (2017) found that increasing space allowance from 0.75 to 1.50 m^2^/ewe significantly increased the time of lying and decreased eating periods, standing time, displacement, and aggressive behavior. Furthermore, increasing the space allowance to 2.25 m^2^/ewe had no effect on the studied parameters. This alteration in behaviors is due to the barrier effect, which hinders the free movement of ewes within the enclosure. Also, it affects locomotion patterns to be a shorter step in small spaces. Also, in small floor spaces, ewes showed fewer resting periods and a higher number of changing positions and frequency of social interactions due to the high disturbance rate. In mothers with newborns, Broster et al. (2012) found that Merino ewes stocked in high SD spent more time with their newborns than those raised in low SD.

It is also important to note that the limited space negatively affects human-sheep relations. For instance, the reaction of tightly housed sheep (0.5 m^2^/head) to humans was less than sheep raised in larger space (Kim et al. 1994). Also, Boissy et al. (2005) showed that restrained sheep from different breeds or crosses respond differently to human contact. Furthermore, females were more active and avoided human-contact more than males. These examples guide us in providing the proper environment for each sheep breed.

### Interaction between SD and housing system influences sheep behavior

The interaction between the housing system and SD is critical and must be considered so as to enhance sheep productivity. In this regard, Caroprese et al. (2009) investigated the effects of the interaction between the housing system and SD (high, 1.5 m^2^/head vs. low, 3 m^2^/head) on the welfare of ewes (BW 54 kg). The housing conditions tested included treatment 1 (1.5 m^2^/ head), treatment 2 (3 m^2^/head), and treatment 3 (3 m^2^/head, 1.5 m^2^/head indoors + 1.5 m^2^/head outdoor). The larger space allowance (3 m^2^) and housing condition (ample yard) in the treatment 3 improved self-grooming, standing, and eating. While it decreased allogrooming (0.04 vs. 0.07 and 0.11%) and ruminating (15 vs*.* 18.4 and 18.6%).

In the outdoor system, Casamassima et al. (2001) found that early-lactating Comisana ewes had a significantly lower idling rate than the indoor ones. Also, they found that outdoor ewes spent more time walking and had higher locomotor activities than indoor ones; this is due to a higher available space and possibly to a stronger kinetic drive to investigate the environment.

In a semi-open barn, Norouzian (2017) studied the effect of three different space allowances on the behavior of Balouchi lambs (35 days old and 6.7 kg BW) for 42 days. Comparing the behaviors of lambs reared in the small floor space (0.34 m^2^/lamb), lambs reared in either medium or large spaces (0.48 and 0.63 m^2^/lamb) significantly spent more time eating, ruminating, walking, playing, and resting. They suggested that a lesser space allowance could prevent animals from lying, which reduces their well-being and productivity. But different space allowances did not significantly affect drinking and urination behaviors.

Regarding the litter/floor types, Chiumenti (1987) suggested slightly greater space allowances, 0.9–1.2 m^2^/head on straw litter, 0.8–1 m^2^/head on the slatted floor, and 2 m^2^ paddock area/head. In addition, Vik et al. (2017) studied the effect of space allowance (0.75, 1.5, and 2.25 m^2^/head) and floor type (straw bedding and expanded metal flooring) on the activity, lying position, and aggressive interactions of Nor-X pregnant ewes. They found that flooring type did not affect the general activities, but ewes in the straw bedding spent more time lying than ewes in the other treatment.

### Interaction between SD and group size affects sheep behavior

Effects of interaction between the SD and group size on sheep behaviors have been reported. For example, Kim et al. (1994) observed that less tightly stocked sheep in small groups moved more than tightly stocked sheep in larger groups. They also noted that tightly stocked sheep (less than 0.5 m^2^/head) had lower overt responses and reactions to humans than those that had less tightly stocked.

In Brazil, Leme et al. (2013) studied the effect of group size (2 or 10) on the behavior of male Santa Ines lambs (average age is 90 days and 20 kg BW) in a limited space (2.4 m^2^/head). They found a higher percentage of lambs housed in double pens remained standing compared to those housed in the collective ones. The eating activity of lambs in the doublepen was 6.9% more than those raised in the collective pens (21%) while ruminating activity in both groups did not differ.

In context, Costa et al. (2019) evaluated the effect of SD on the ingestive behavior of Santa Inês male lambs (age of 4 months and 21 kg BW) housed individually or in a double stall. The male lambs were stocked in the two types of covered stalls: double stalls (two male lambs/stalls) of 3 m^2^ and individual stalls of 1.5 m^2^. Lambs housed in the individual stalls spent more ruminating time (583 min/day) and less eating time than those raised in the double stalls (503 min/day). Animals housed in the individual stalls showed higher feeding efficiency (FE) (691.57 vs. 381.62 g dry matter intake/min). Lambs also spent more time in idleness. Lambs of the double stalls consumed more water, which resulted in increasing urination. However, this activity did not interfere with their total weight gain. The findings of Leme et al. (2013) and Costa et al. (2019) referred to the feeding behavior of lambs in groups is enhanced by social facilitation, which results in a higher feed intake (FI) than this of individually stocked lambs.

On the other hand, Ruiz-de-la-Torre and Manteca (1999) studied the effect of SD, low (1 head/m^2^) and high (3.3 head/m^2^), of male and female prepubertal lambs of 20 kg BW on social behavior. Results indicated that social mixing conditions decreased the number of aggressive interactions between males and females (including head-to-head clashes, head-to-body butting, and mountings). Finally, the proper space allowance should fit the size and status of animal. For instance, Loynes (1983) suggested that a minimum space allowance of 0.60 m^2^/head for lambs (15–25 kg BW), and be increased to 1 m^2^/head for the heavier ones (25–40 kg BW).

It is noteworthy that sheep from different pure breeds (Romanov, ROM and Lacaune, LAC), the two F1 crossbreeds (RL and LR), and the offspring of ewes from these four genotypes sired with Berrichon-du-Cher rams (BCF) responded differently to the same environmental stimuli (Boissy et al. 2005). For example, they found BCF crossbreds had more locomotion activity and attempt to escape than purebreds and F1.

The specific response of each breed is unclear, but it may be due to selection for behavioral characteristics or association with breeding programs for a type of production (direct additive genetic). Also, the genetic background effect on sheep response and their behaviors has been notified by Engeldal et al. (2013) and Polat and El Sabry (2014). On contrary, Boissy et al. (2005) found that the maternal effect on the behavior is non-significant. In this regard, we suggest that a part of the response of sheep to environmental stimuli might be due to the epigenetic effect as found by El Sabry and Tzschentke (2010). In the following, Sheep specific behaviors that are influenced by space allowance are summarized in Table 1.

**Table 1:** The effect of stocking density on species-specific behaviors of sheep

Social behavior	Category	High SD	Low SD	References
Eating time, ruminating	Lamb, dairy sheep	↓↑	↑↓	Caroprese et al. (2009), Avero’s et al. (2014), Vik et al. (2017),Norouzian (2017)
Drinking	Dairy sheep, lamb	No	No	Caroprese et al. (2009), Norouzian (2017)
Lying time, walking	Lamb, dry and dairy sheep	↓	↑	Bøe et al. (2006), Caroprese et al. (2009), Vik et al. (2017), Norouzian (2017)
Standing, displacement, aberrant	Ram, dry and dairy sheep	↑	↓	Dalholt (1985), Bøe et al. (2006), Engeldal et al. (2013), Vik et al. (2017), Norouzian (2017)
Locomotion, playing, resting, exploratory	Lamb, ram, dairy sheep	↓	↑	Kim et al. (1994), Engeldal et al. (2013), Avero’s et al. (2014), Vik et al. (2017), Norouzian (2017)
Aggressive, pushing	Ram, dairy sheep	↑	↓	Kim et al. (1994), Caroprese et al. (2009), Engeldal et al. (2013), Vik et al. (2017)
Self-care	Dairy sheep	↓	↑	Caroprese et al. (2009)
Allogrooming	Dairy sheep	↑	↓	Caroprese et al. (2009)

↑Increase, ↓decrease, and *No.* no significant effect

### Effects of SD on meat production of sheep

It is known that space limitations can adversely affect the welfare of animals (Estevez 2007) and their performances. Regardless of housing type, indoor or outdoor, the growth performance of lambs is associated with space allowance (Hodge et al. 1991), where lambs stocked at 0.33 m^2^ per head significantly lose more weight and had lower FI and FE than those housed at 1.5 m^2^/head.

In growing lambs, Gonyou et al. (1985) found that reducing space allowance from 0.52 to 0.3 m^2^/lamb resulted in about 10% less average daily gain (ADG), where the optimal ADG is 350–450 g that can be achieved at space allowance rate of 0.5 m^2^/lamb.

These findings are partially in line with those of Norouzian (2017), who studied the effect of three different space allowances on the growth performance of Balouchi lambs (35 days age and 6.7 kg BW) for 42 days in summer. Lambs kept in large pens (0.63 m^2^/lamb) showed an increase in FI compared to those in the small (0.34 m^2^/lamb) or medium (0.48 m^2^/lamb) pens. However, the ADG, feed conversion rate (FCR), and BW were affected by space allowance. This result is due to the high locomotor activity of the lambs housed in medium and large spaces.

In the grazing system, in Brazil, Leme et al. (2013) studied the effect of group size (2 or 10) on the ADG of Santa Ines male lambs (age 90 days and 20 kg BW) in a limited space (2.4 m^2^/head). Lambs housed in double pens had a higher FI and ADG than those housed in the collective pens, which is due to social facilitation that increased eating activity in the double pen by 6.9%. Moreover, we suggest that in collective pens, the FI of lambs was lower due to the higher competition for accessing fodder.

In castrated Romney Marsh lambs, Rovira (2013) recorded that the low SD group (10/ha) had higher ADG (206 g/day) and final BW (50.8 kg) than the high SD group (24/ha) that had ADG of 40 g/day and BW of 37.5 kg. They indicated that the SD range from 10 to16 lambs/ha is recommended for fattening lambs.

In the indoor system, Horton et al. (1991) observed that reducing the space allowance from 0.99 to 0.62 m^2^/lamb impaired both FI and ADG of Dorset lambs by 11% and 14%, respectively. These results could be due to the high cortisol level in the blood of lambs of the smaller space group. Similarly, El Sabry and Almasri (2022) mentioned high blood cortisol levels of calves that are kept in small spaces. Also, the live performance parameters of female lambs, including ADG, weaning weight, and days to slaughter were more affected by higher SD. Also, Earle et al. (2017) found that the increase in the days to slaughter (171, 184, and 189 days) in different breeds (Suffolk and Belclare-sired crossbred ewes) were significantly associated with the SD (10, 12, and 14 ewe lambs/ha). While carcass weight was slightly affected by SD, being the highest (19.9 kg) in low SD compared to 19.8 and 19.7 in middle and high SD.

Regarding group size, Leme et al. (2013) showed that group size (2 or 10) in a limited space (2.4 m^2^/head) did not affect ADG. Similarly, Costa et al. (2019) found that lambs in either individual stalls of 1.50 m^2^ or double stalls (two males/stall of 3.0 m^2^) had similar FI, ADG, final BW, and daily water intake.

It is a worth noting that although increasing space allowance led to an increase in FI, there was no increase in BW and ADG of lambs. This is due to the increase in the required maintenance for animals that are housed in high space allowance vs*.* those that were housed in the smaller space.

## Effects of SD on milk yield of sheep

Milk production is a complicated and stressful physiological process that makes animals sensitive to exogenous stressors. Optimal space allowance is an important environmental variable that is positively correlated with milk yield. For example, Sevi et al. (1999) found that the space allowance of 2 m^2^/head positively impacted milk yield and milk content of protein, casein, and fats, compared to the negative effects of smaller space allowances of 1 or 1.5m^2^/head.

Regarding the interaction between SD and the housing system, In Italy, Caroprese et al. (2009) reported that Comisana sheep kept in either space allowance of 3 m^2^/head or 1.5 m^2^ indoor + 1.5 m^2^ outdoor produced more milk (973 and 979 g/day, respectively) than sheep reared in an indoor (1.5 m^2^/head, 787 g/day), whereas, somatic cell count as a quality marker was higher in the milk of sheep stocked in smaller floor space of 1.5 m^2^/head, which attributed to larger space allowance and the availability of outdoor areas that can improve the welfare and production performance of the lactating ewe. On the other hand, Casamassima et al. (2001) showed that housing systems, either indoor (1.8 m^2^/ewe) or outdoor (10 m^2^/ewe), did not affect milk composition and production of early-lactating Comisana ewes. Thus, it can be suggested that a space allowance of 2–3 m^2^ for a lactating ewe can positively affect milk quality and quantity.

## Effects of SD on wool production

Generally, both wool quantity and quality are affected by nutrition, SD, and dietary feed additives (Yeates et al. 1975). In context, White and McConchie (1976) found a decline in the fleece weight and the quality parameters of wool (smaller fiber diameter, shorter staple, and more staple crimp frequency) due to the increase in SD from 4.9 to 12.4 Merino sheep /ha. Similarly, Brown (1977) reported that greasy wool production and fiber diameter decreased as the SD increased. Also, under a rotational grazing system, George and Pearse (1978) and Carey et al. (1988) studied the effect of stocking SD (8, 12, and 16 Merino ewes /ha) and (2, 4, and 6 Corriedale ewes/ha) on wool quality and production. They found that increasing the SD tended to reduce wool production and its quality parameters, e.g., staple length and fiber diameter. While Carey et al. (1988) found that the SD did not affect the greasy fleece weights for Corriedale ewes.

## Effects of SD on reproductive traits of sheep

The stress, which results from small floor space, can adversely affect the reproduction performance of different farm animals such as sheep (Holmøy et al. 2012), cattle (Miranda-de la Lama et al. 2013), buffalo (El Sabry and Almasri 2022), quail (El Sabry et al. 2022) and goats (El Sabry and Almasri 2023).

It is worth noting that the birth weight and survival rate for lambs from multiple lambing are more sensitive to high SD than ones from single lambing, inducing a 50% higher mortality rate (Donnelly 1984). Also, Robertson et al. (2012) found that the high SD of 30 ewes/ha reduced the survival of lambs born alive by 24% compared to those at the low stocking of 16 ewes/ha. However, they noticed that lamb birth weight, marking weight, and ewes BW were not affected by stocking rates.

Alliston and Lucas (1979) did not observe any significant difference in Black Welsh Mountain ewe reproductive performance when comparing outdoor rearing to housing for all or part of the winter in the UK. In context, Kleemann et al. (2006) reported that the survival rate of either single or twin lambs was not affected by different SD when ranging between 2.9 and 23.9 commercial Merino ewes/ha in Australia. This result agreed with the findings of Earle et al. (2017), who found that the lambing difficulty, type of birth, birth weight, and the number of lambs weaned per ewes were not affected by different stocking rates (10, 12, and 14 ewes/ha).

## Effects of SD on the immune response of sheep

Stocking density plays a critical role in sheep’s immunity. In this context, Caroprese et al. (2009) indicated that the sheep housed in a greater space allowance of 3 m^2^/ewe had a higher humoral immune response than the ewes stocked at 1.5 m^2^/ewe. In addition, free access to an outdoor area increased the cell-mediated immune response of ewes compared to enclosure indoors. It can be suggested that this incompetency of immune response can be due to the chronic stress of over stocking throughout the experimental time. Finally, the effects of SD on wool, meat, milk production, reproduction traits, and immunity are summarized in Table 2.

**Table 2:** Effect of stocking density on meat, milk and wool production, and reproductive traits of sheep

Studied parameters	Low SD	High SD	References
Wool quantity and quality	↑	↓	White and McConchie (1976), George and Pearse (1978), and Carey et al. (1988)
Meat yield, average daily gain, carcass weight	↑	↓	Davies and Southey (2001), Rovira (2013), and Earle et al. (2017)
Milk production	↑	↓	Sevi et al. (1999)
Twining rate and survival rate,	↑	↓	Davies and Southey (2001), Robertson et al. (2012)
Immunity	↑	↓	Caroprese et al. (2009)

↑Increase, ↓decrease

### Suggested methods for mitigating the adverse effects of high stocking rate

Previous studies indicated the deleterious influences of a high SD on sheep’s productive and reproductive performances (Engeldal et al. 2013). In south-central Australia, Kleemann et al. (2006) studied the effects of the group size and SD, among other factors, on the reproductive traits of commercial Marino sheep. They found that the survival rate of lambs was curvilinearly related to flock size and not SD. It was suggested that optimization of management factors could enhance the reproductive traits and lambs’ BW, e.g., survival rate of lambs increased with the optimum flock size at 400 ewes.

Also, Rovira (2013) reported that adding grain supplementation or expanding grazing time improves the ADG and final BW of tightly stocked fatting Merino ewe lambs (high SD, 16–24 head/ha) under un-supplement grazing to be like those of the low SD stocked lambs (10–16 head/ha) that kept under the unsupplemented system. Ruiz-de-la-Torre and Manteca (1999) suggested that social mixing between males and females may alleviate the stress of SD and reduce aggressive behavior.

## Conclusion

This review identified a wide range of documented examples of the effect of SD on the welfare indices and productive traits of sheep. According to available information, it can be concluded that.

- Interaction among SD, group size and housing system should be considered for assigning the optimal SD for meat, milk, and wool-type sheep. As well as, sheep breeds respond differently to the SD alteration, so the space allowance requirement for different breeds should be revised.
- Increasing space allowance and accessibility to outdoor yard improve the yield and quality of milk, and welfare indices of lactating ewes. Moreover, prolonging grazing time, adding grains, and social mixing could be practical solutions for reducing aggressive and agonistic behaviors, and improving productivity of sheep herds.
- Considering an economic-welfare balance, the suggested space allowances ranges are: 0.48–1 m^2^/head for lambs, 1.6–2.5 m^2^/head for a ram, and 3 m^2^/head for a ewe (1.5 m^2^ indoor + free access to1.5 m^2^ outdoor).

## Data Availability

Not applicable.
